# Tobacco and the Doctor

**Published:** 1909-10

**Authors:** 


### Tobacco and the Doctor.

Lately it was publicly announced that the Academy of Medicine of Cincinnati would devote the evening of April 20 to the discussion of tobacco and its effect on the human system. When we think of the amount of tobacco that is raised in the United States; the large sums of money invested in the tobacco trade; the revenue it pays to the Government, and its immense consumption; and the still unsettled question whether it is good or bad for those who use it, we get a suggestion of the importance of the subject. More money is spent for tobacco than is spent for bread, and there are a great many people in this country who would not exchange a cigar for a meal, even when they are hungry.

Naturally, much interest arose when it became known that tobacco was to be discussed by so learned a body as the Cincinnati Academy of Medicine, and not only the result of the discussion, but the facts and arguments by which that result might be reached were eagerly awaited.

Doctors are known to accomplish much of their good work by withholding from their patients and the public the true inwardness of their treatment; they write prescriptions in mysterious characters, and talk either not at all or in learned technical phrases which are not understood by the uninitiated. Experience confirms the wisdom of this policy in general, and it is seldom relaxed, so that if one wishes a little inside information, he has to resort to some strategy to get it; and for this reason a stenographer was induced to hide himself within hearing on that occasion, and to take down verbatim all that was said.

Three papers were read, each by a learned and well-known member of the medical profession, discussing the effects of tobacco on the eyes, the heart and the nerves, respectively.

The writers were like Martin Van Buren in politics, they were thoroughly non-committal, but they brought out many facts and some theories. If anything, they rather leaned to the sentiment that tobacco, like M. L. Conway's idea about sin, was not so very bad if moderately indulged in.

In the general discussion which followed much cleverness was developed and some bitterness. Of course, there was every degree of approval and dissent from the views of the respective essayists. If Solomon thinks that in a multitude of counselors there is safety, it involves necessarily some agreement between them. In their debate the views of the learned members of the Academy seemed to get farther and farther apart, and when the wordy war grew hot so many were talking at the same time that the stenographer was unable to follow any one speaker; but could catch only a word or a part of a sentence here and there, as it arose above the general din; and some parts of his notes, literally written out, read this way:

"The learned doctor—Mr. President—nobody thinks—nervous system—ninety years—coffin—solace of tired—smoke while children starve—Mail Pouch—old colored man—cigarettes—preposterous idea—blessings to humanity—Murad poison—aid digestion —coffin nails—prolong life—nervous wreck—poverty—blessing of old age—American Tobacco Company—iron nail plug—fragrant aroma—destructive of tissues—from Havana duty free," and so on, getting louder every moment until the chairman's gavel came down with a bang, with calls to order. When quiet was restored, the president said:

"You know, gentlemen, that the rule in the Academy is that not more than two men may speak at the same time. When five are talking, as was the case just now, it is impossible to follow any one, and there is nothing but hopeless confusion. Now, let us begin again. Every man shall have his opportunity. The question is, 'What's the use of tobacco, anyway?' Dr. Saddlebags has the floor."

That learned doctor argued that it is only a noxious weed which should be uprooted; rooted out and burned in the fires of Gehenna, and all recollection of it banished from the earth. He said that America had blessed the world by furnishing it with the wild turkey, the unostentatious white potato, Indian corn, and Peruvian bark to cure chills, but there were two things that America had contributed to the world which had done more harm than all the uncounted wealth of the western continent could pay for; one was tobacco and the other was the pox.

This remark brought out both cheers of approval and groans of deep dissent. One man fairly screamed that it was a sin to speak of tobacco and the pox in the same breath, and he hoped that God would forgive him for having done so. He protested against putting the blessings and the cursings of humanity in the same catalogue, but the doctor proceeded in the same strain.

"Did you not read," said he, "in the versatile and loquacious *Commercial Tribune* only a few days ago how a man down in Texas refused to eat, sleep or drink for five days and nights, but was incessantly smoking cigarettes?" and he quoted that case to show that tobacco had befuddled the man's wits and was a demoralizing poison, but he was answered by the suggestion that possibly the man would have died but for his love of smoking and his ambition to prolong his life to continue to enjoy the weed as long as possible.

Such interruptions did not deflect the doughty doctor the breadth of a hair from his line of argument, and he kept right on until he had exhausted the vocabulary of abuse and denunciation, and had proven, or thought he had proven, that tobacco is worse than all the snake bites in the world, a hell-invented plant, born to all bad ends and no good ones, and he concluded by saying that if it were possible for him to believe that God Almighty would create and let loose in the world any specific evil for the purpose of doing the greatest possible harm to mankind. He had done so when He permitted tobacco to have an existence among herbs of the field.

Several doctors joined in the debate, the most of them being dead against the weed, and so outspoken were they that the few apologists who spoke in its defense seemed to make very little impression.

Tobacco was arraigned, and seemed to appeal in vain for a champion to plead its cause, but as is so often the upshot in story books, at the last moment the devil's advocate shied his castor into the ring.

He was a man of years, slender and keen, with bright eyes and sharp symmetrical features, a clear voice and a steadiness of nerve which would naturally characterize a man who for many years had been a skillful and daring surgeon. He conciliated his auditors by gentle tones and showed no inclination to wrangle and after giving them a placebo, he said something like this:

"When you doctors come in after a hard day's work, tired and fagged in body and mind, some of you seek rest by taking half a grain of morphine; some of you dope yourselves with what is sold to the common world as a sleeping powder; others take cocaine; some take wine; some black coffee, and some a bottle of beer; and some even send out the growler and bring it in by the bucketful. Now, when I get a chance, after my dinner, to retire to my library and to needed rest, I light a cigar. The slow dreamy puffing rests me; the curling white smoke, shedding the soothing fragrance of a real Havana, refreshes me; on the fleecy clouds hope and I walk in dreams; I build my castles like a boy; I am consoled and comforted; I rest and renew my youth; I pass the night in healthful slumber, and I greet the rising sun with a blessing on tobacco in my heart."

And here, when the speaker had so interested his auditors that every man without exception was wrapped in eager attention, he raised his left hand, between whose fingers he held a fat cigar—a fine one, with a bright, handsome belly-band around its middle, and he addressed to it these lines:

TO A CIGAR.

Yes, social friend, I love thee well,
In learned doctors' spite;
Thy clouds all other clouds dispel,
And lap me in delight.

By thee, they cry, with phizzes long,
My years are sooner passed.
Well, take my answer, right or wrong,
They's sweeter while they last.

And oft, mild friend, to me thou art
A monitor, though still;
Thou speak'st a lesson to my heart
Beyond the preacher's skill.

Thou'rt like the man of worth who gives
To goodness every day;
The odor of whose virtue lives
When he has passed away.

Oft as thy snowy column grows,
Then breaks and falls away;
I trace how mighty realms thus rose,
Then tumbled to decay.

And what is he who smokes thee new?
A little moving heap;
That soon like thee to Fate must bow—
With thee in dust must sleep.

But though thy ashes downward go,
Thy essence rolls on high;
Thus when my body must lie low
My soul shall cleave the sky.

The venerable physician resumed his seat in the midst of a silence which indicated that, after all, the devil had found an advocate.

The debate was ended and the question called for. The chairman stated it: "What's the use of tobacco, anyway?" and asked all who were in favor of the question to say "Aye." There was a roar of ayes.

Then: "All who are contrary minded signify it by saying 'No'!" There was a roar of noes.

The chairman said he was not able to say which had it, and he called for a rising vote.

"All who are in favor of the question will please rise and stand until they are counted."

Every man in the room stood up and they were counted.

"Gentlemen, please be seated. Now, all those opposed will please rise and stand until they are counted."

Again every man in the room rose to his feet and they were all again counted.

The chairman announced a tie vote; but his decision was immediately appealed from. It was denied that it was a tie vote, but a case in which every man had voted on both sides.

The chairman then, much perplexed, asked the Academy to remain in abeyance a few moments while he collected his thoughts; he said he was not able to state exactly where they were.

The suspense was becoming oppressive, when the old surgeon—the devil's advocate—arose, holding a large sheet of paper before him.

He said that it would be best to consider the question just voted on as settled, and he begged to offer a resolution which he thought would show the way out. He presented it as follows:

"Resolved, That the Academy of Medicine of Cincinnati thinks that girls, boys and women should not use tobacco in any form; that all men who can use it with impunity may do so as much as they like, if they can afford it; that all men whom it injures should abstain from it altogether."

And he moved its adoption.

There was a chorus of seconds.

And the chairman put it, "All who are in favor of the resolution offered by Dr. O'Connor, of Connersville, please say 'Aye.' "

There was a roar of ayes.

"All opposed please say 'No.' "

No graveyard was ever more silent than the room after that call.

But the chairman did not seem to be convinced, and so he called on all who were in favor of the resolution to rise and be counted.

All stood up, and were counted and resumed their seats.

"All opposed please rise and stand until counted."

Every mother's son of them appeared to be glued to his chair.

Then the wearied chairman announced that the resolution was adopted. The Academy adjourned.—D. G. A. in *Albright's Office Practitioner.*

